# Koch’s Treatment of Tuberculosis

**Published:** 1891-01

**Authors:** 


### KOCH'S TREATMENT OF TUBERCULOSIS.

THE uncertainty which has followed this treatment from the beginning still remains, and, possibly, it will continue to be a subject of mingled doubt and interest to the medical profession and of painful expectancy to the afflicted for some time to come.

The reports received from Berlin have not been encouraging, but it is to be hoped that the question may reach a settlement at an early period; and this result is all the more likely to be attained, since the area of clinical observation has been widened and transferred to the critical tests of the entire medical world.

The report of "The Tuberculosis Commission" of the University of Pennsylvania has just been issued in the form of a bulletin in the *University Medical Magazine.* This contains a full summary of the clinical results achieved up to date, December 15.

The report says, "In regard to the nature of the new remedy, there is absolutely nothing known."

It is shown to be a very dangerous remedy, as the report confirms the general statement that "cases in the late stages of pulmonary consumption are not suited to this treatment." So far as is now known, infections practised upon such patients are liable to result fatally. In view of the fact that the remedy has now reached many localities on this side, it cannot be long before definite results will be arrived at, though it may take several years to determine the question in all its relations.

This whole matter has so thoroughly and so properly claimed the interest of the medical profession that the more serious ethical side has, apparently, not been taken into consideration.

This may be stated in the form of a query: Has the medical profession abandoned the safe ground of learning everything in regard to the origin and testing all articles introduced before adopting them in practice? The answer would, doubtless, be given in the negative; yet the evidence would go far to indicate that the professions and practice of the past had not been lived up to in the present instance.

There may be a difference as wide as the ocean in regard to the origin and value of a proprietary remedy and that discovered by Koch; but the consistency that would denounce the one as empirical and make use of the other, a secret preparation, does not impress itself strongly on the average mind.

It is to be regretted that public clamor should have influenced to a departure from the old and safe mode of open explanation and thorough testing in private before public announcement. As the case stands, there is sure to be disappointment and chagrin in store for those who advised and adopted the present course. The motive which actuated Dr. Koch in keeping the preparation a secret does honor to him as a man, but it is unfortunate for his reputation and that of the entire medical profession that it was adopted.

